# Clinical Consequences and Molecular Bases of Low Fibrinogen Levels

**DOI:** 10.3390/ijms19010192

**Published:** 2018-01-08

**Authors:** Marguerite Neerman-Arbez, Alessandro Casini

**Affiliations:** 1 Department of Genetic Medicine and Development, Faculty of Medicine, University of Geneva, 1211 Geneva, Switzerland; 2 Division of Angiology and Hemostasis, Faculty of Medicine, Geneva University Hospitals, 1211 Geneva, Switzerland; alessandro.casini@hcuge.ch

**Keywords:** fibrinogen, genetics, bleeding, thrombosis, women’s health, zebrafish

## Abstract

The study of inherited fibrinogen disorders, characterized by extensive allelic heterogeneity, allows the association of defined mutations with specific defects providing significant insight into the location of functionally important sites in fibrinogen and fibrin. Since the identification of the first causative mutation for congenital afibrinogenemia, studies have elucidated the underlying molecular pathophysiology of numerous causative mutations leading to fibrinogen deficiency, developed cell-based and animal models to study human fibrinogen disorders, and further explored the clinical consequences of absent, low, or dysfunctional fibrinogen. Since qualitative disorders are addressed by another review in this special issue, this review will focus on quantitative disorders and will discuss their diagnosis, clinical features, molecular bases, and introduce new models to study the phenotypic consequences of fibrinogen deficiency.

## 1. Introduction

**Figure 1:**
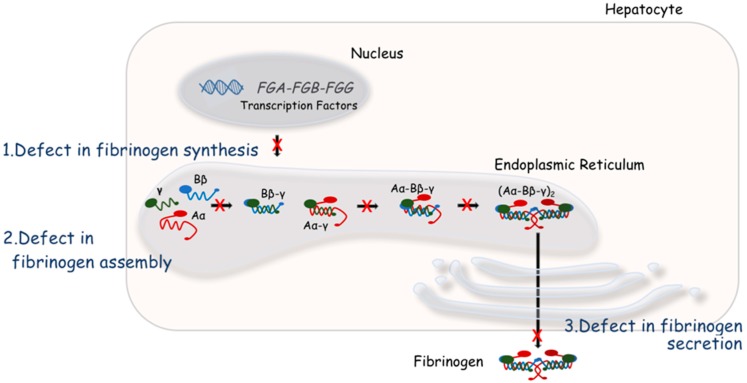
Fibrinogen synthesis in hepatocytes. Fibrinogen synthesis is regulated by both transcriptional and translational mechanisms. After individual fibrinogen chains are translated, fibrinogen assembly proceeds in the lumen of the endoplasmic reticulum in a stepwise manner. Single chains are first assembled to form Bβ-γ and Aα-γ intermediates, then Aα-Bβ-γ half-molecules, and finally the hexameric glycoprotein (Aα-Bβγ)_2_. Quantitative fibrinogen disorders can result from defects in synthesis, assembly, or secretion of fibrinogen in circulation [3].

The ultimate goal of the coagulation cascade is the controlled conversion of fibrinogen into fibrin, an insoluble polymer which gives structural stability, strength, and adhesive surfaces to growing blood clots. Human fibrinogen is produced in the liver from three homologous polypeptide chains, Bβ, Aα, and γ, encoded by the fibrinogen gene cluster *FGB*, *FGA*, and *FGG* on human chromosome 4. Two copies of each polypeptide chain assemble in a stepwise manner (Figure 1) to form a 340 kD hexamer (AαBβγ)_2_ held together by disulfide bonds. Normal plasma fibrinogen levels vary between 1.5 and 3.5 g/L. Fibrinogen and fibrin play major roles in multiple biological processes in addition to hemostasis and thrombosis, i.e. fibrinolysis where the fibrin clot is broken down; matrix physiology by interacting with FXIII, plasminogen, fibronectin, and vitronectin; wound healing, inflammation, infection, cell migration, cell-cell interactions, and angiogenesis; and tumor growth and metastasis [1].

Inherited disorders of fibrinogen are rare. Quantitative (also known as Type I) disorders affect the quantity of fibrinogen in circulation: hypofibrinogenemia is characterized by fibrinogen levels lower than 1.5 g/L; afibrinogenemia, an autosomal recessive disease, is characterized by the complete deficiency of fibrinogen. Qualitative (also known as Type II) disorders affect the quality of circulating fibrinogen: in dysfibrinogenemia, an autosomal dominant disease, fibrinogen antigen levels are normal, while in hypodysfibrinogenemia levels are reduced. The study of inherited fibrinogen disorders, characterized by extensive allelic heterogeneity, allows the association of defined mutations with specific defects, providing significant insight into the location of functionally important sites in fibrinogen and fibrin. Since the identification of the first causative mutation for congenital afibrinogenemia in 1999 [2], our group and others have elucidated the underlying molecular pathophysiology of numerous causative mutations leading to fibrinogen deficiency, developed cell-based and animal models to study human fibrinogen disorders, and further explored the clinical consequences of absent, low, or dysfunctional fibrinogen. Since qualitative disorders are addressed by another review in this special issue, this review will focus on quantitative disorders.

## 2. Diagnosis of Quantitative Fibrinogen Disorders

The diagnosis of afibrinogenemia is based on standard clotting assays (i.e., activated partial thromboplastin time, prothrombin time, thrombin time, reptilase time) and fibrinogen assessment [4]. All tests depending on the fibrin clot as an end-point are infinitely prolonged [5] and fibrinogen is undetectable by both functional (i.e., Clauss assay, prothrombin time-derived test) and antigenic (i.e., enzyme-linked immunoabsorbent assays, radial diffusion, precipitation, thrombin clotting) methods [6]. In hypofibrinogenemia, standard clotting assays are variably prolonged according to the circulating fibrinogen level. Functional and antigenic fibrinogen levels are proportionally decreased [7]. A ratio of functional on antigenic fibrinogen level higher than 0.7 is traditionally used to distinguish between hypofibrinogenemia and dysfibrinogenemia [8], although the sensitivity and specificity of this cut-off has never been established [9]. The amplitude of coagulation curves from thrombin time tests should allow for the easy determination of the type of fibrinogen deficiency when the antigenic fibrinogen assessment is not available [10]. Due to the limited sensitivity of coagulation assays in the case of very low fibrinogen levels (e.g., <0.5 g/L), it can be challenging to distinguish afibrinogenemia from severe hypofibrinogenemia [11]. Global hemostasis assays such as thromboelastography can provide information on the ability to restore clot formation after fibrinogen replacement [12,13].

## 3. Clinical Features

The severity and pattern of clinical manifestations are dependent on the levels of fibrinogen. In afibrinogenemia bleeding is the main symptom, whereas in hypofibrinogenemia patients are more frequently asymptomatic. Some clinical features are specifically related to the type of quantitative fibrinogen disorders, as described in detail below.

### 3.1. Bleeding

The basal coagulant fibrinogen level is strongly correlated with the bleeding phenotype [14]. In a retrospective study from the European Network of Rare Bleeding Disorders, including 46 patients with fibrinogen deficiency, a mean fibrinogen activity level of at least 0.7 g/L protected from spontaneous bleeding, while patients with a level above 1.0 g/L did not experience bleeds [15]. Data on the epidemiology of the bleeding complications are scarce. From a retrospective survey study on 100 patients with quantitative fibrinogen disorders, the mean annual incidence of bleeding episodes was 0.5 for patients on prophylactic replacement therapy (range 0–2.6) and 0.7 for patients on on-demand therapy (range 0–16.5) [16]. As indicated in Table 1, umbilical cord bleeding is a common symptom in afibrinogenemia [17]. Muscle hematoma, oral cavity bleeding, post-surgical bleeding, and skin hematoma are also frequent [18]. Central nervous system bleeding, often recurrent and in young children, is the main causes of morbidity and mortality [19,20]. Hemarthroses are less frequent and less invalidating than in patients with hemophilia [21,22].

Hypofibrinogenemic patients are often asymptomatic and diagnosed during routine laboratory testing, before surgery, or in the setting of familial explorations [23]. In symptomatic patients, the bleeding phenotype is usually linked to trauma, surgery [24], or concomitant coagulopathies [25].

### 3.2. Thrombosis

Thrombosis in both venous and arterial sites are a typical complication of afibrinogenemia [26]. The pathogenesis of thrombus formation has not been elucidated. Even in the absence of fibrinogen, patients are still able to generate thrombin, both in the initial phase of limited production and in the secondary burst of thrombin generation [27], as suggested by an increase in prothrombin activation fragments and thrombin-antithrombin complexes [28]. Thrombin, when not trapped within the fibrin clot [29], is available for platelet activation and smooth muscle cell migration and proliferation [30] and leads to large and loosely packed platelet thrombi [31,32]. The absence of fibrin, with the decrease of its anti-thrombin effect, could lead to increased levels of circulating thrombin [29,30]. This hypothesis is challenged by observations in which circulating thrombin generation is decreased in the absence of fibrin, as thrombin bound to fibrin is protected from irreversible inactivation by antithrombin [33]. Even if fibrinogen replacement has often been indicated as a risk factor, there is no definite evidence that a well-controlled fibrinogen supplementation increases the risk of thrombosis [34]. Thrombotic events can be recurrent, especially in the arterial peripheral territory, despite an accurate antithrombotic regimen [35]. Co-existing plasma hypercoagulability, inherited or acquired, may contribute to the patient’s overall thrombotic phenotype [36]. A defect in the fibrinolytic system may also be a contributing factor [29].

Thromboses are less frequent in hypofibrinogenemia. The low levels of circulating fibrinogen are probably enough to counteract the induced thrombotic phenotype observed in afibrinogenemia. It should be noted that low fibrinogen levels do not compensate a hypercoagulable state [37,38].

### 3.3. Specific Symptoms

Some clinical manifestations are specific to the type of quantitative fibrinogen disorder [23]. Bone pains have been reported in afibrinogenemic patients [42]. These symptoms are related to bone cysts comparable to the pseudo-tumors observed in patients with hemophilia [43]. They often develop during childhood and are primarily located in the diaphysis of long bones. The etiology is unknown, but partial improvement observed under fibrinogen replacement suggests hemorrhage during bone remodeling [44]. Spontaneous splenic rupture can occur in afibrinogenemic patients and should be considered in the case of brutal onset of abdominal pain [45]. The underlying mechanism is undetermined [46]. Even though a conservative approach has been successfully reported, surgical management is probably more suitable to avoid clinical adverse outcomes in case of recurrent rupture [47].

Hypofibrinogenemic patients can suffer from fibrinogen storage disease, which is due to the accumulation of fibrinogen aggregates in the endoplasmic reticulum of hepatocytes [48]. The mechanism by which hepatic intracellular inclusions escape the highly regulated cellular clearance machinery is not clear [49]. A defect in the endoplasmic reticulum-associated degradation pathway leading to autophagy saturation may be a contributing factor [50]. Usually, patients have moderate hypofibrinogenemia with a liver disease of varying severity [51,52]. The diagnosis requires hepatic biopsy followed by imaging to reveal intracellular inclusions and liver histology with positive immunostaining for fibrinogen [53].

### 3.4. Women’s Health

Women with quantitative fibrinogen disorders are more prone to complications, especially during the childbearing period. In afibrinogenemia, more than 50% of women suffer from menorrhagia (Table 1) [54]. Peritoneal bleeding secondary to hemorrhagic rupture of ovarian cysts is common [55]. Afibrinogenemic pregnant women are at risk of several obstetrical complications, from spontaneous abortion to placental abruption, including postpartum hemorrhage [56]. Indeed, fibrinogen is necessary to provide placental integrity by supporting the spreading of cytotrophoblasts at 4–6 weeks of gestation [57] and for the optimal development of the fetal-maternal vascular throughout pregnancy [58]. A well-dosed fibrinogen replacement regimen is necessary for all afibrinogenemic women to maintain pregnancy to term, as well as to avoid placental abruption and post-partum hemorrhage [59,60].

Depending on the fibrinogen levels, hypofibrinogenemic women are also affected by menorrhagia [61] and obstetrical complications [62] such as recurrent placenta abruption, suggesting insufficient fibrinogen to sustain placenta development [63].

## 4. Molecular Bases of Quantitative Fibrinogen Disorders

Fibrinogen is synthesized in hepatocytes and secreted in the form of a hexamer (AαBβγ)_2_ (Figure 1). The three genes encoding fibrinogen; Bβ (*FGB*), Aα (*FGA*), and γ (*FGG*), are clustered in a region encompassing 50 kilobases on human chromosome 4 [64]. *FGA* and *FGG* are transcribed from the reverse strand, in the opposite direction to *FGB*. Each gene is separately transcribed and translated to produce nascent polypeptides of 644 amino acids (Aα), 491 amino acids (Bβ), and 437 amino acids (γ). Alternative splicing for *FGA* produces an extended isoform (Aα-E, present in only 1–2% of circulating fibrinogen), while alternative splicing of *FGG* produces the γ’ isoform, present in 8–12% of circulating fibrinogen in the heterodimeric form (AαBβγAαBβγ’) [65].

Congenital fibrinogen disorders are caused by mutations in one of the three fibrinogen chain-encoding genes. While the first dysfibrinogenemia mutation was identified as early as 1968 [66] using amino acid composition analysis of thrombin-digestion products of purified patient fibrinogen, before the genomic sequences of the three fibrinogen genes were determined, the molecular basis of afibrinogenemia was elucidated much later. The first causative mutation for congenital afibrinogenemia, a large recurrent deletion in *FGA*, was identified in a Swiss family in 1999 [2]. Since this time, more than 200 different mutations have been identified as accounting for afibrinogenemia or hypofibrinogenemia. This research has already led to improved diagnosis including prenatal diagnosis, genetic counselling, and care of the patients affected by these diseases.

Several recent reviews and mutation updates are available [4,23,67] in addition to a Fibrinogen Mutation Database (Available online: www.geht.org) [68]. Database entries compiling data from published manuscripts, conference abstracts, and online submissions contain information on the type of disorder when known (i.e., afibrinogenemia, hypofibrinogenemia, dysfibrinogenemia, hypodysfibrinogenemia, fibrinogen storage disease, amyloidosis), patient origin if available, as well as presence of bleeding and/or thrombotic symptoms [68]. The latest release (July 2017, release 43) of this important open-access resource lists 775 entries corresponding to 366 distinct mutations; 200 of these are associated with quantitative disorders (including six distinct mutations for fibrinogen storage disorders, all in *FGG*), 90 with qualitative disorders, and 18 with renal amyloidosis (all in *FGA*). The remaining mutations are associated with discordant phenotypes in different patients, or the disorder is not stated. The distribution of mutations shows 143 distinct mutations in *FGA* (73 quantitative, 32 qualitative, 18 amyloidosis mutations), 87 in *FGB* (61 quantitative, 18 qualitative), and 136 in *FGG* (66 quantitative, 40 qualitative).

Polymerase chain reaction amplification of exons and intron-exon junctions of the three fibrinogen encoding genes, followed by sequencing, allows the identification of the vast majority of causative mutations for fibrinogen disorders. While high-throughput sequencing techniques such as whole exome sequencing are now readily available, this strategy is still the most time- and cost-effective approach for the majority of cases.

For afibrinogenemia and hypofibrinogenemia, causative mutations can be divided into two main classes: null mutations with no protein production at all and mutations producing abnormal protein chains which are retained inside the cell [4] (Figure 1). Null mutations, i.e., large deletions, frameshift, early-truncating nonsense, or splice-site mutations, account for the majority of afibrinogenemia alleles, as expected given the complete deficiency of fibrinogen in circulation. Hypofibrinogenemia is generally caused by heterozygosity for these mutations. Interestingly, missense mutations can also lead to fibrinogen deficiency. These are clustered in the highly conserved C-terminal globular domains of the Bβ and γ chains [4]. Functional studies of these mutations in transfected cells have demonstrated either impaired assembly and secretion [69,70] or normal assembly but impaired secretion [71], demonstrating the importance of these globular structures in the quality control of fibrinogen biosynthesis [72]. The common Aα chain encoded by *FGA* does not contain a globular domain in its C-terminus, but rather a flexible coil where missense mutations do not severely impact hexamer assembly and secretion, and therefore can be present in the circulating fibrinogen of dysfibrinogenemia patients.

Most mutations can be identified using a PCR-sequencing screening strategy. The identification of larger, more complex rearrangements such as the recurrent *FGA* 11 kb deletion requires other techniques, such as Southern blotting, genomic sequencing, or tailored PCR analysis [73]. To date, only four large deletions (greater than 1 kb), all involving *FGA*, have been reported. As described above, an interesting subset of patients with hypofibrinogenemia has accompanying liver fibrinogen storage disease characterized by endoplasmic reticulum fibrinogen-positive liver inclusions. Only six mutations in *FGG*, all occurring within or near polymerization pocket hole “a”, are known so far to cause hepatic storage disease [74,75,76,77,78].

## 5. New Models to Study Phenotypic Consequences of Fibrinogen Deficiency

Studies aiming to determine the underlying molecular mechanism by which causative mutations lead to fibrinogen deficiency have been performed in various transfected cell models. This is because fibrinogen is synthesized in hepatocytes, and patient liver biopsies are rarely available to assess the quantity and quality of fibrinogen transcripts, individual chains, folding intermediates, and hexamers. This approach has allowed to functionally characterize defects in mRNA expression, mRNA splicing, fibrinogen assembly, and fibrinogen secretion. However, these simple models cannot fully explore the underlying molecular bases of this pathology and explain the resulting complex clinical manifestations described above.

The zebrafish, *Danio rerio*, is increasingly used to model human diseases (reviewed for example by Gut et al. [79]). The zebrafish has several advantages: the optical clarity of embryos and accessibility of cells and tissues, the rapid development of homologous vertebrate organs, the large amount of progeny, and the availability of several techniques for genome editing and manipulation of gene expression, e.g., CRISPR-Cas9 for knockouts, transient mRNA expression and morpholinos for knockdowns. The zebrafish genome is highly conserved, with 70% of human genes having a zebrafish ortholog. Zebrafish can be used for forward genetic screens as well as small molecule chemical screens which can translate into new treatments for human disorders.

Zebrafish have very rapid and microscopically transparent embryogenesis that is well-suited for in vivo studies. Their development occurs after the external fertilization of eggs and, in contrast to rodent vascular biology models, the blood vessels of zebrafish larvae are clearly visible during embryogenesis between 48 hours and 5 days. Blood hemostasis resembles the mammalian system [80,81]. While zebrafish, like birds, have nucleated thrombocytes rather than platelets, these are functionally similar since platelet receptors are found on thrombocytes and mammalian platelet agonists lead to thrombocyte aggregation. The majority of human coagulation factors present in zebrafish are single copy and show high conservation [82,83]. In particular, the three zebrafish fibrinogen genes are all localized on chromosome 1, although the compact configuration observed for the human fibrinogen cluster is not conserved with zebrafish *FGG* located approximately 15 Mb away from *FGA* and *FGB* with two intervening genes.

Targeted gene knockdown experiments have been performed in zebrafish using antisense morpholino oligonucleotides for a number of coagulation factors including prothrombin [84], factor VII [85], vWF [86], and fibrinogen [87]. In this latter study, fibrinogen combined morpholino knockdown fish showed intraventricular and intramuscular hemorrhage [87]. The use of morpholinos can, however, result in phenotype variability due to incomplete gene silencing. Our group produced a zebrafish model of afibrinogenemia with a bleeding phenotype similar to the human phenotype, by the targeted mutation of the *FGA* gene using zinc-finger nuclease technology [88]. Using a panel of anti-zebrafish fibrinogen antibodies, fibrinogen was undetectable in the plasma of homozygous mutant fish. These fish showed unprompted bleeding at various sites with cephalic and ventral hemorrhaging, as well as reduced survival compared with control animals. Following this study, the first transmissible zebrafish model of a defined human bleeding disorder, a zebrafish model of anti-thrombin III deficiency, was also produced using zinc finger nuclease mutagenesis [89]. Here, homozygous mutant zebrafish embryos had spontaneous venous thrombosis, resulting in early adulthood lethality. In 2017, the genome editing of Factor X in zebrafish was achieved, resulting in a major hemostatic defect in zebrafish embryos and widespread hemorrhage at later stages [90].

Importantly, in addition to the simple observation of spontaneous bleeding and/or thrombotic phenotypes in zebrafish embryos and adults, several quantitative functional assays relevant to the study of hemostasis and thrombosis have been developed [91,92,93].

Laser-mediated endothelial injury of the posterior caudal vein and the dorsal aorta of zebrafish larvae have been successfully used to assess thrombus formation under different experimental conditions. Various parameters of clot formation, stability, and dissolution can be monitored in mutant zebrafish compared to wildtype animals. In our laboratory and others, this model is now used to study the impact of mutations in coagulation factors that result in deficient fibrin formation or platelet function [88,90].

## 6. Conclusions and Perspectives

The establishment of strong translational research environments combining clinical data and long-term patient follow-up with more fundamental laboratory approaches has led to major progress in the understanding of fibrinogen disorders, both qualitative and quantitative. Even though the number of cases studied is already quite substantial, the collection and comparison of molecular, biochemical, and clinical data continues to yield valuable information on the development and course of these diseases as well as on the choice of the most appropriate treatments. This research has already significantly accelerated diagnosis, enabled genetic counselling and prenatal diagnosis, and improved care of the patients affected by these diseases. Zebrafish models will also contribute to a greater understanding of the bleeding and thrombotic phenotypes of patients with fibrinogen disorders, and may open new avenues of research for therapeutic approaches.

## Figures and Tables

**Table 1:** Clinical manifestations reported in selected series of afibrinogenemic patients.

Author	Patients, *n*	Males, *n* (%)	Umbilical Bleeding, *n* (%)	Muscle Bleeding, *n* (%)	Joint Bleeding, *n* (%)	CNS Bleeding, *n* (%)	Oral Cavity *, *n* (%)	Menorrhagia, *n* (% **)	Skin, *n* (%)	Miscellaneous ***, *n* (%)
Lak [21]	55	27 (49)	45 (85)	40 (72)	30 (55)	3 (10)	40 (72)	14 (50)	NA	23 (40)
Monaldini [39]	6	3 (50)	0 (0)	2 (33)	1 (17)	1 (17)	3 (50)	0 (0)	1 (17)	0 (0)
de Moerloose [18]	110	45 (41)	66 (60)	51 (46)	33 (30)	22 (20)	32 (29)	36 (55)	51 (46)	39 (36)
Sumitha [40]	20	12 (60)	13 (65)	0 (0)	1 (5)	5 (25)	7 (35)	3 (38)	17 (85)	6 (30)
Asselta [41] ****	13	5 (38)	8 (62)	0 (0)	0 (0)	1 (8)	8 (62)	1 (8)	8 (62)	6 (46)
Nagler [26]	4	3 (75)	2 (50)	4 (100)	4 (100)	2 (50)	2 (50)	1 (100)	2 (50)	1 (25)

*: including gingival bleeding and epistaxis; **: % of women; ***: including hematuria, post-surgery, retroperitoneal, gastro-intestinal bleeding, and hemoptysis; ****: clinical data available for 10 patients, CNS: central nervous system.
